# Metformin regulates atrial SK2 and SK3 expression through inhibiting the PKC/ERK signaling pathway in type 2 diabetic rats

**DOI:** 10.1186/s12872-018-0950-x

**Published:** 2018-12-13

**Authors:** Chang-He Liu, Na Hua, Xi Fu, Yi-Long Pan, Bin Li, Xiao-Dong Li

**Affiliations:** 1 Department of Cardiology, Shengjing Hospital of China Medical University, No. 36 Sanhao Street, Heping District, Shenyang, 110004 People’s Republic of China; 2 Department of Otolaryngology, Affiliated Zhongshan Hospital of Dalian University, Dalian, 116001 People’s Republic of China

**Keywords:** Diabetes mellitus, Metformin, SK channels, PKC, ERK, Atrial fibrillation

## Abstract

**Background:**

Our previous study showed that metformin regulates the mRNA and protein levels of type 2 small conductance calcium-activated potassium channel (SK2) and type 3 small conductance calcium-activated potassium channels (SK3) in atrial tissue as well as the ion current of atrial myocytes in rats with type 2 diabetes mellitus (T2DM), but the underlying signaling mechanism is unknown. This study aimed to investigate whether metformin regulates atrial SK2 and SK3 protein expression in T2DM rats though the protein kinase C (PKC)/extracellular signal-regulated kinase (ERK) signaling pathway.

**Methods:**

A T2DM rat model was established using a high-fat and high-sugar diet combined with a low-dose intraperitoneal injection of streptozotocin (STZ). The rats were randomly divided into the following five groups: the control group, the untreated T2DM group, the metformin-treated only group, the phorbol 12-myristate 13-acetate (PMA; a PKC agonist administered by intraperitoneal injection) treatment group, and the recombinant human epidermal growth factor (rh-EGF; an ERK agonist administered by tail vein injection) treatment group. The activity of PKC in atrial tissues was assayed by a PKC kinase activity assay kit. The protein expression of SK2, SK3, and phosphorylated ERK (pERK) were determined by western blotting and immunohistochemistry.

**Results:**

Compared with the Control group, atrial PKC activity and pERK and SK3 protein expression were increased, while SK2 protein expression was decreased in atrial tissues of T2DM rats. Eight weeks of metformin treatment inhibited the PKC activity and pERK and SK3 expression, and elevated SK2 expression compared with the T2DM group. Compared with the metformin-treated only group, the injection of rh-EGF increased pERK and SK3 expression, and decreased SK2 expression; the injection of PMA increased PKC activity and SK3 expression, and decreased SK2 expression. In addition, the injection with PMA significantly elevated the expression of pERK.

**Conclusions:**

The PKC/ERK signaling pathway is involved in the downregulation of SK2 expression and the upregulation of SK3 expression in the atrium of T2DM rats. Long-term metformin treatment prevents the SK2 downregulation and the SK3 upregulation through inhibiting the PKC/ERK signaling pathway.

**Electronic supplementary material:**

The online version of this article (10.1186/s12872-018-0950-x) contains supplementary material, which is available to authorized users.

## Background

Type 2 diabetes mellitus (T2DM) is prevalent worldwide and causes a variety of metabolic disorders (e.g., hyperglycemia, hyperlipidemia, and insulin resistance) and cardiovascular complications and increases the risk of atrial arrhythmias such as atrial fibrillation (AF) [1, 2]. Recent studies have shown that metformin, as the first-line medicine for T2DM, reduces the incidence of AF [3]. Studies have shown that atrial ion channel remodeling is the basis of AF. However, few studies have investigated the effect of metformin on the remodeling of atrial ion channels in DM, and the molecular mechanism is unknown.

Small conductance calcium-activated potassium channels (SK channels) as a kind of outward calcium-sensitive potassium channel has three subtypes: SK1, SK2, and SK3. In the cardiovascular system, SK channels are primarily distributed in atrial myocytes and play an important role in myocardial repolarization [4]. Recent studies have shown that SK channels are closely associated with AF [5]. Our previous study showed that metformin regulates the mRNA and protein levels of atrial SK2 and SK3 as well as the ion current of atrial myocytes in T2DM rats [6], but the signaling mechanism is unknown.

The protein kinase C (PKC)/extracellular signal-regulated kinase (ERK) signaling pathway, which is extensively expressed throughout the body, is involved in the inflammatory response, collagen synthesis, and apoptosis and plays an important role in the regulation of cardiac electrophysiology and function [7, 8]. In the case of DM, factors such as hyperglycemia, insulin resistance, oxidative stress, and non-enzymatic glycosylation activate the PKC/ERK signaling pathway [9, 10], whereas metformin plays a protective role by inhibiting activation of the PKC/ERK signaling pathway and blocking the release of inflammatory mediators [11, 12]. However, additional research is needed to determine whether the PKC/ERK signaling pathway is involved in the changes in and metformin regulation of atrial SK2 and SK3 ion channels in T2DM rats.

This study was designed to determine whether metformin regulates atrial SK2 and SK3 in T2DM rats via the PKC/ERK signaling pathway to provide a potential theoretical basis for a mechanism of atrial ion channel remodeling in DM and the improvement of diabetes-induced AF.

## Methods

### Animals

Age-matched, male Wistar rats weighting 250–300 g were purchased from Huafukang Bioscience CO.INC (Beijing, China), and housed with adequate food and water at an ambient temperature (22 °C ± 2 °C) and relative humidity (55% ± 5%), with a 12:12 h light:dark cycle. The experiment protocol conformed to the Guide for the Care and Use of Laboratory Animals by the US National Institutes of Health (NIH Publication No. 85–23).

The rats were randomly divided into two groups: the control group (Con) rats were given a normal diet, while the experimental group rats were given a high-fat and high-sugar diet (10% lard, 20% sucrose, 1.0% bile acids, 2.5% cholesterol, and 66.5% standard diet) [13]. Four weeks later, after fasting for 12 h (water was allowed), the experimental group rats were given a single intraperitoneal injection of streptozotocin (STZ; 30 mg/kg; Sigma-Aldrich, St Louis, MO, USA) in citrate buffer (pH = 4.5) [14]. Three days later, after fasting overnight, an Accu-Chek glucometer (Roche, Mannheim, Germany) was used to collect a blood sample from the tail vein of each rat to determine blood glucose levels. The model establishment was considered successful with fasting blood glucose (FBG) ≥11.1 mmol/L [15], and the rats continued to receive the high-fat and high-sugar diet. Four weeks later, after overnight fasting, a blood sample from the tail vein was measured to determine the blood glucose level and fasting insulin (FINS) and to calculate the insulin sensitivity index (ISI). Moreover, intraperitoneal glucose tolerance was assessed. Briefly, fasting blood glucose was measured from the tail vein after overnight fasting. Then rats were intraperitoneally injected with 50% glucose (2.0 g/kg, in 1.5 mL saline), and blood glucose was immediately measured at 30-, 60-, 90-, 120-min with Accu-Chek glucometer (Roche). Insulin resistance was determined by ISI and intraperitoneal glucose tolerance. Rats with an FBG ≥ 11.1 mmol/L and an increased insulin resistance were used for the T2DM model in this study [16]. The control group rats were given an intraperitoneal injection of an equal volume of citrate buffer, and a blood sample was collected from the tail vein to determine FBG and FINS simultaneously. All animals were monitored for blood glucose and body weight weekly.

All T2DM rat models were randomly divided into two groups: the untreated T2DM group (DM) and the metformin treatment group (Met). The Met group was given 300 mg/kg/d metformin (via gavage) for 8 weeks [17, 18], while the Con and the DM groups were given an equal volume of saline (via gavage) for 8 weeks. During the last two weeks, the Met group was further randomly divided into three groups: metformin-treated only (Met), the phorbol 12-myristate 13-acetate (PMA, a PKC agonist) treatment group (PMA), and the recombinant human epidermal growth factor (rh-EGF, an ERK agonist) treatment group (EGF). The PMA group rats were given a intraperitoneal injection of 2 μg/kg PMA (Abcam, Cambridge, UK) in dimethylsulfoxide (DMSO; Santa Cruz Biotech, Santa Cruz, CA, USA) every other day for two weeks [19]; the EGF group was given a tail vein injection of 10 μg/kg rh-EGF (R&D Systems, Minneapolis, MN, USA) in distilled water every day for two weeks [20]; and the Con, DM, and Met groups were injected with an equal volume of the same liquid. Body weight, FBG, and FINS were monitored again before the rats were sacrificed by intraperitoneal injection with 100 mg/kg pentobarbital sodium, and the atrial tissues were rapidly isolated after the sacrifice.

### PKC activity

A PKC kinase activity assay kit (Abcam) was used to assess PKC activity. Briefly, the lysate of rat atrial tissue was added to a PKC-specific substrate-coated microplate, after which ATP was added to phosphorylate PKC to initiate substrate phosphorylation. After the kinase reaction was stopped, a specific antibody against the phosphorylated substrate was added to the microplate, and after washing, a horseradish peroxidase (HRP)-labeled secondary antibody was added. Tetramethylbenzidine (TMB) was used for staining at room temperature, and then the reaction was terminated. The intensity of TMB staining was proportional to the PKC activity in the sample. A microplate reader was used to determine the absorbance value at 450 nm, and the relative PKC activity was calculated and expressed in comparison to the control group which was set to ‘1’.

### Western blotting

The expression levels of SK2, SK3, and pERK proteins in atrial tissues of rats were assayed by western blotting according to a previously described method [21]. Anti-SK2 (1:1000; Invitrogen, Carlsbad, CA, USA), anti-SK3 (1:1000; Invitrogen), anti-pERK (1:1000; Cell Signaling Technology, Danvers, MA, USA), and anti-GAPDH (1:5000; Proteintech, Chicago, IL, USA) primary antibodies were used to incubate the membranes. The bands were visualized with enhanced chemiluminescence (ECL) assay, and the intensity was measured using ImageJ software (NIH, Bethesda, MD, USA). The relative protein expression was expressed as the ratio of the band intensity normalized to GAPDH and compared with that of the control group.

### Immunohistochemistry

The expression levels of SK2 and SK3 proteins in atrial tissues of rats were verified by immunohistochemistry (IHC). Briefly, the isolated rat atrial tissues were fixed in paraformaldehyde and sectioned. The samples were treated with 3% hydrogen peroxide (H2O2) and pre-incubated with 10% goat serum, and then incubated with anti-SK2 (1:100; Invitrogen) and anti-SK3 (1:100; Invitrogen) primary antibodies overnight at 4 °C. Subsequently, the samples were incubated with biotin-labeled secondary antibody and streptavidin-horseradish peroxidase (SA-HRP). Diaminobenzidine (DAB) was used for color reaction, and the color development was monitored under a microscope. Images were collected using the NIS-Elements F3.0 software (Nikon, Tokyo, Japan) under a light microscope at 400× magnification. The mean optical density (MOD) values of IHC images were measured using image-pro plus software v6.0 (Media Cybernetics, Inc., Silver Spring, MD, USA) by single-blind method.

### Statistical analysis

ImageJ (NIH) was used to analyze the band intensity. Excel 2013 (Microsoft Corporation, Redmond, WA, USA) was used to process the data. SPSS v19.0 (SPSS Inc., Chicago, IL, USA) was used for statistical analysis. GraphPad Prizm v5.0 (GraphPad Software, Inc., La Jolla, CA, USA) was used to export the images. All the data are expressed as mean ± SE. Comparison of data before and after modeling was done with independent sample t-test. One-way analysis of variance (ANOVA) was performed for multiple comparisons, followed by Fisher’s least significant difference test. *P* < 0.05 was considered significant.

## Results

### The characteristics of T2DM rat models and the hypoglycemic effect of metformin

**Fig. 1:**
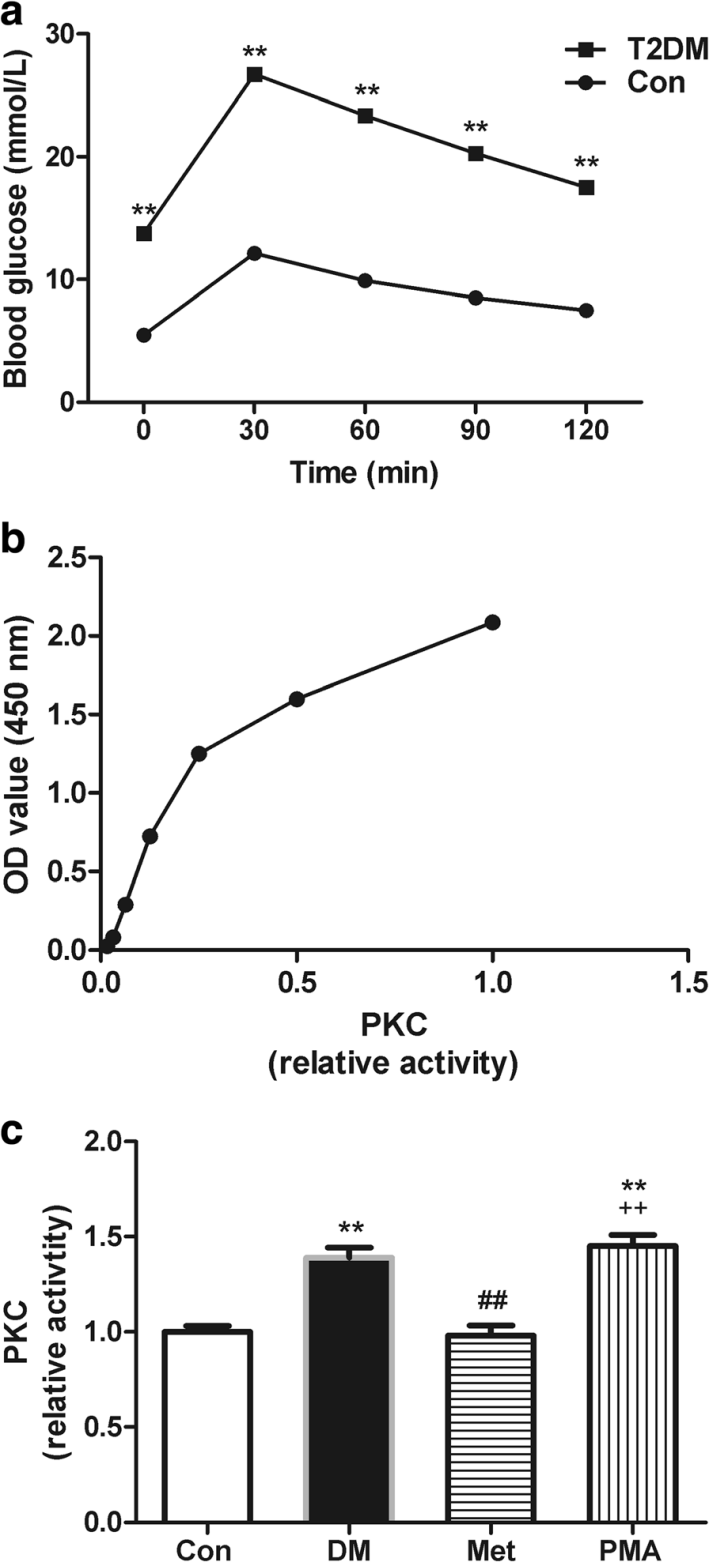
Blood glucose levels and the standard curve and the relative PKC activity in the atria of rats from the Con, DM, Met, and PMA groups. **a** Blood glucose levels during intraperitoneal glucose tolerance test in control and T2DM rats. **b** The standard curve for the PKC activity assay. **c** Relative histogram of relative PKC activity demonstrates the effects of T2DM, 8-week metformin treatment as well as 2-week injection of PMA on the PKC activity in rat atria among the four groups (*n* = 5). ^**^P < 0.01 vs Con; ^##^*P* < 0.01 vs DM; ^++^*P* < 0.01 vs Met

Table 1 shows the characteristics of T2DM rat models induced by an eight-week high-fat and high-sugar diet combined with intraperitoneal injection of low-dose STZ (30 mg/kg). We observed that the body weights (BW) significantly decreased, the FBG and FINS significantly increased, and the calculated ISI value significantly decreased in diabetic rats compared with those of the rats in the Con group. Figure 1a shows a significant elevation of blood glucose levels during the intraperitoneal glucose tolerance test in diabetic rats. These results indicated that the diabetic rats had typical characteristics of T2DM, such as blood glucose elevation, glucose tolerance impairment, and insulin resistance. In addition, we also observed that metformin significantly reduced the FBG level of T2DM rats at the end of eight-week treatment (14.64 ± 0.61 vs 10.96 ± 0.31, *P* < 0.01; *n* = 8).

**Table 1:** General characteristics of rats in Con and T2DM

group	n	BW (g)	FBG (mmol/L)	FINS (mIU/L)	ISI
Con	8	408.00 ± 5.89	5.55 ± 0.08	19.21 ± 0.91	- 4.66 ± 0.06
T2DM	32	358.25 ± 3.35^**^	14.01 ± 0.41^**^	25.41 ± 0.54^**^	- 5.86 ± 0.04^**^

*BW* body weight, *FBG* fasting blood glucose, *FINS* fasting insulin, *ISI* insulin sensitivity index, calculated as ln [(1/(FBG × FINS)]

^**^P < 0.01 vs Con

### Metformin prevented SK2 downregulation and SK3 upregulation by inhibiting atrial PKC activity in the atrial tissue of T2DM rats

**Fig. 2:**
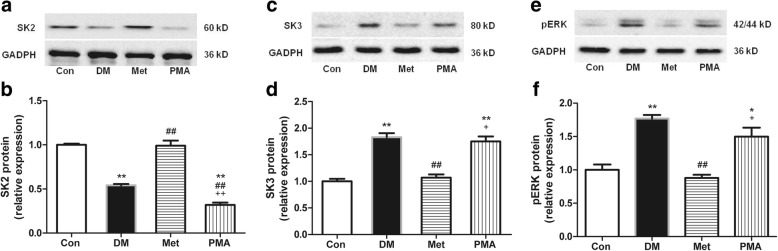
The relative expression of SK2, SK3, and pERK proteins in the atria of rats from the Con, DM, Met, and PMA groups. Representative Western blots [SK2 (**a**), SK3 (**c**), and pERK (**e**)] and relative histograms [SK2 (**b**), SK3 (**d**), and pERK (**f**)] demonstrate the effects of T2DM, 8-week metformin treatment as well as 2-week injection of PMA on the expression of SK2, SK3, and pERK in rat atria among the four groups (*n* = 4–5). ^*^P < 0.05, ^**^P < 0.01 vs Con; ^##^*P* < 0.01 vs DM; ^+^*P* < 0.05, ^++^*P* < 0.01 vs Met

**Fig. 3:**
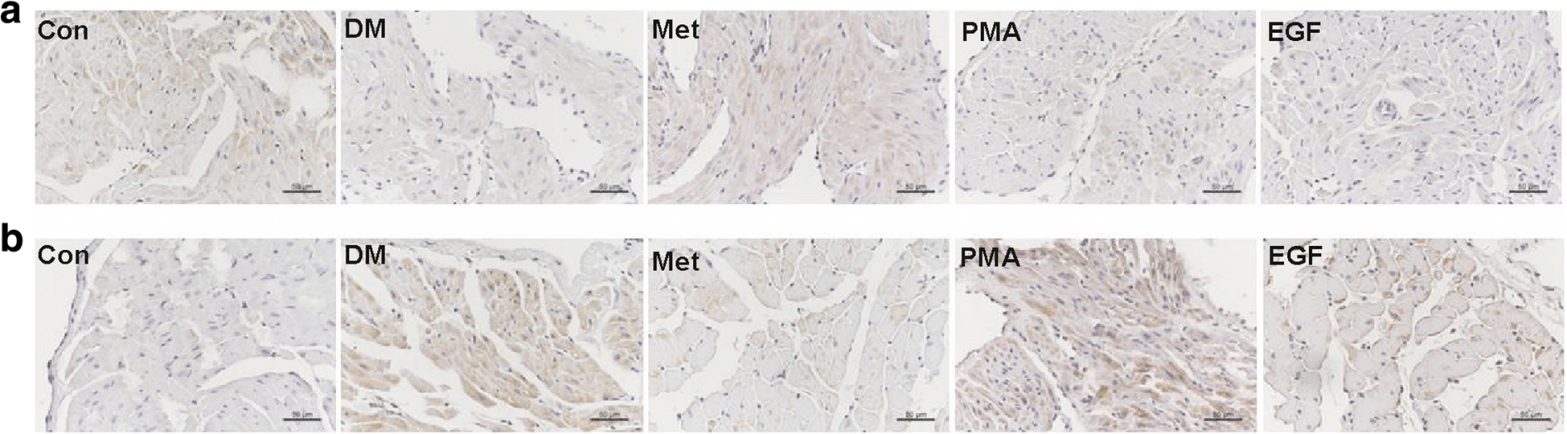
Typical images of immunohistochemistry [SK2 (**a**), and SK3 (**b**)] in the atria of rats from the Con, DM, Met, PMA, and EGF groups. SK2 and SK3 were stained brownish yellow, and the nuclei were stained blue. The intensity of the brownish yellow color represents the SK2 and SK3 expression levels (scale bar = 50 μm)

A PKC kinase activity assay kit was used to determine atrial PKC activity in the atrial tissues of rats from the Con, DM, Met, and PMA groups. In addition, western blotting was performed to assess the expression of SK2, SK3, and pERK proteins in these groups. The results showed that the PKC activity was 40% higher (Fig. 1c, *P* = 0.009; Additional file 1: Table S1), meanwhile, the SK2 expression was 56% lower (Fig. 2b, *P* < 0.001; Additional file 1: Table S2) and the SK3 expression was 83% higher (Fig. 2d, *P* = 0.004; Additional file 1: Table S3) in the DM group than that observed in the Con group. Compared with the DM group, 8 weeks of metformin treatment inhibited the PKC activity by 30% (Fig. 1c, *P* = 0.006), meanwhile, increased SK2 expression by 83% (Fig. 2b, *P* < 0.001) and decreased SK3 expression by 42% (Fig. 2d, *P* = 0.007) in the Met group. To further confirm that metformin regulated the expression of SK2 and SK3 by inhibiting the PKC activity, PMA (a PKC agonist) was injected in the PMA group rats. The results showed that PMA significantly PKC activity by 48% (Fig. 1c, *P* = 0.003). In addition to the increase in PKC activity, we found a significant decrease of 68% in SK2 expression (Fig. 2b, *P* < 0.001) and a significant increase of 64% in SK3 expression (Fig. 2d, *P* = 0.013). Therefore, these data suggested that prevented SK2 downregulation and SK3 upregulation by inhibiting PKC activity. In addition, immunohistochemistry was performed to detect the expression of SK2 and SK3 in each group. We also found that SK2 expression was decreased (*P* = 0.002) and SK3 expression was increased (*P* < 0.001) in the DM group relative to the Con group. The 8-week metformin treatment elevated SK2 expression (*P* = 0.014) and reduced SK3 expression (*P* < 0.001). The PKC agonist PMA downregulated the SK2 expression (*P* = 0.002) and upregulated the SK3 expression (*P* = 0.001) (Fig. 3; Additional file 1: Table S5 and Table S6).

### Metformin prevented SK2 downregulation and SK3 upregulation by inhibiting pERK expression in the atrial tissue of T2DM rats

**Fig. 4:**
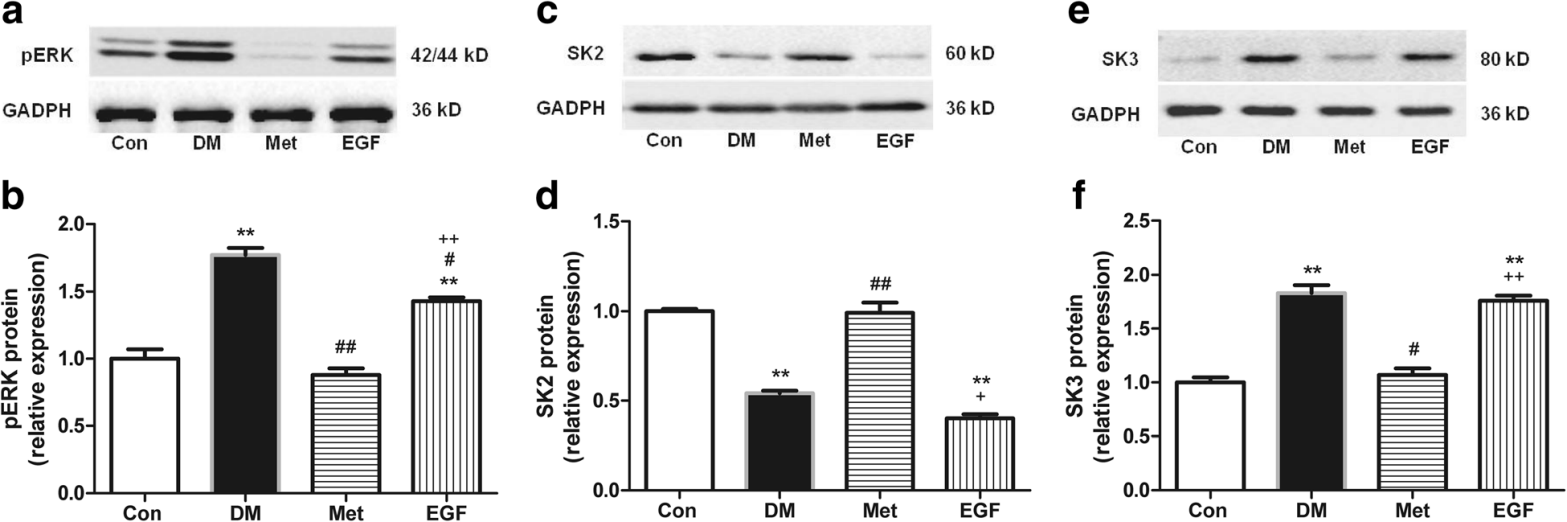
The relative expression of pERK, SK2, and SK3 proteins in the atria of rats from the Con, DM, Met, and EGF groups. Representative Western blots [pERK (**a**), SK2 (**c**), and SK3 (**e**)] and relative histograms [pERK (**b**), SK2 (**d**), and SK3 (**f**)] demonstrate the effects of T2DM, 8-week metformin treatment as well as 2-week injection of rh-EGF on the expression of pERK, SK2, and SK3 in rat atria among the four groups (*n* = 4–5). ^**^P < 0.01 vs Con; ^#^*P* < 0.05, ^##^*P* < 0.01 vs DM; ^+^*P* < 0.05, ^++^*P* < 0.01 vs Met

Western blotting was performed to assess the expression of pERK, SK2, and SK3 proteins in the atrial tissues of rats from the Con, DM, Met, and EGF groups. The results showed that the pERK expression was 77% higher (Fig. 4b, *P* < 0.001; Additional file 1: Table S4), meanwhile, the expression of SK2 was reduced (Fig. 4d, *P* < 0.001; Additional file 1: Table S2) and the expression of SK3 was increased (Fig. 4f, *P* = 0.001; Additional file 1: Table S3) in the DM group than that observed in the Con group. Compared with the DM group, 8 weeks of metformin treatment inhibited the expression of pERK by 50% (Fig. 4b, *P* < 0.001), meanwhile, increased SK2 expression (Fig. 4d, *P* < 0.001) and decreased SK3 expression (Fig. 4f, *P* = 0.002) in the Met group. To further confirm that metformin regulated the expression of SK2 and SK3 by inhibiting pERK expression, rh-EGF (an ERK agonist) was injected in the EGF group rats. The results showed that rh-EGF significantly increased pERK expression by 63% (Fig. 4b, *P* = 0.001). In addition to the increase in pERK expression, we found a significant decrease of 60% in SK2 expression (Fig. 4d, *P* < 0.001) and a significant increase of 64% in SK3 expression (Fig. 4f, *P* = 0.003). Therefore, these data suggested that metformin prevented SK2 downregulation and SK3 upregulation by inhibiting pERK expression. In addition, immunohistochemistry was performed to detect the expression of SK2 and SK3 in each group. We also observed the ERK agonist rh-EGF downregulated SK2 expression (*P* = 0.008) and upregulated SK3 expression (*P* = 0.018) (Fig. 3; Additional file 1: Table S5 and Table S6).

### Metformin inhibited atrial PKC activity and downregulated pERK expression in the atrial tissue of T2DM rats

In addition to atrial PKC activity in the Con, DM, Met and PMA groups, we analyzed pERK expression in each group by Western blotting. The results showed that both PKC activity (Fig. 1c; Additional file 1: Table S1) and pERK expression (Fig. 2f; Additional file 1: Table S4) were significantly higher in the DM group than in the Con group. After 8 weeks of metformin treatment, both PKC activity (Fig. 1c) and pERK expression (Fig. 2f) were significantly lower in the Met group than in the DM group. To confirm that metformin inhibited pERK expression by inhibiting PKC activity, PMA (a PKC agonist) was injected in the PMA group rats. The results showed that PKC activity was enhanced by 48% (Fig. 1c, *P* = 0.003), meanwhile, pERK expression was increased by 70% in the PMA group than in the Met group (Fig. 2f, *P* = 0.010), further demonstrating that ERK is a downstream signaling molecule of PKC and that metformin regulates the expression of atrial SK2 and SK3 in T2DM rats via the PKC/ERK signaling pathway.

## Discussion

In this study, we aimed to investigate the molecular mechanisms of metformin regulation on SK2 and SK3 ion channels in the atrium of T2DM rats. The following results were obtained: (1) the downregulation of SK2 protein and the upregulation of SK3 protein occurred in the atria of T2DM rats compared with the control group; (2) PKC/ERK signaling pathway mediated the downregulation of SK2 protein and the upregulation of SK3 protein; (3) long-term metformin reversely regulated the expression of SK2 and SK3 proteins through PKC/ERK signaling pathway. To our knowledge, the present study is the first to demonstrate the molecular mechanisms of metformin regulation on the SK2 and SK3 ion channels through inhibiting the PKC/ERK signaling pathway in the atrium of T2DM rats. In this study, we also used metformin to demonstrate the PKC/ERK signaling pathway is involved in the changes in atrial SK2 and SK3 in diabetes for the first time.

### The T2DM rat model

T2DM is a complex polygenic disease characterized by hyperglycemia and insulin resistance [22]. Spontaneous T2DM can occur in some hereditary DM rat models, such as Zucker diabetic fatty fa/fa rats and Goto-Kakizaki rats, which have been extensively used in animal studies. However, these transgenic models are virtually homogeneous with monogenic inheritance, which differs from the heterogeneity of T2DM patients in clinic [23, 24]. A low-dose STZ injection (< 35 mg/kg) damages fewer β-cells and can be used in combination with a long-term high-fat and high-sugar diet to induce insulin resistance and generate T2DM models [25, 26]. Moreover, STZ-induced diabetic models are heterogeneous, as in human diabetic patients. In addition, the model is cost-effective and easier to maintain with a high success rate. We are experienced in using this method to generate the T2DM model, which was used in this study [16]. Both FINS and ISI are measures of insulin resistance. The T2DM rat model used in this study was also characterized by hyperglycemia and insulin resistance.

### Atrial fibrillation, SK channels, and T2DM

AF is a complex functional disorder associated with cardiac electrophysiology. It is one of the primary T2DM-related complications that increases mortality [23, 27]. Currently, it is believed that atrial ion channel remodeling is the primary cause of AF [23]. However, the mechanisms of the remodeling are complex, and how diabetes induces AF remains unclear. As a result, treating AF is one of the clinical difficulties now. Recent studies have shown that remodeling of SK channels is closely associated with AF. SK channels are widely present in cardiomyocytes and are involved in the initiation of action potential. They play an important role in the regulation of myocardial depolarization and the maintenance of normal cardiac electrical activity [4]. SK channels have at least three subtypes, SKl (KCNNl), SK2 (KCNN2), and SK3 (KCNN3). Several studies have shown that low [28] or high [29] SK2 expression and high SK3 expression [30] affect the action potential duration (APD) of cardiomyocytes, resulting in AF. This study observed low SK2 expression and high SK3 expression in the atria of T2DM rats, which was consistent with the findings of our previous study [6]. The three subtypes of SK channels in cardiomyocytes are interrelated and are not completely independent. We believe that complex interactions play a role in the differential expression of the subtypes of SK channels, which would also help to explain the electrophysiological mechanism of how diabetes induces AF.

### Metformin regulates the SK2 and SK3 expression

Several clinical studies have shown that metformin, the most commonly used diabetes medicine, reduces the risk of AF in diabetic patients [3]. It has been shown to play a role in reducing inflammatory response and oxidative stress independent of the hypoglycemic effect [31, 32]. This study showed that after 8 weeks of metformin treatment, SK2 expression was significantly increased, and SK3 expression was significantly decreased in the atria of T2DM rats, which was consistent with our previous findings [6]. The primary target of antiarrhythmic drugs is ion channels, which contribute to action potential formation in the myocardium. SK channels are important ion channels for maintaining and stabilizing the action potential of cardiomyocytes. Metformin alleviates the remodeling of SK2 and SK3 channels under pathological conditions, which partially explains how long-term metformin treatment reduces and prevents AF.

### PKC/ERK signaling pathway is involved in the changes in and metformin regulation of atrial SK2 and SK3 in T2DM rats

Although several studies have revealed the changes in atrial SK channels under diabetic conditions, relatively few studies have investigated the molecular mechanisms. In the present study, we used metformin as a tool to partially explore the molecular mechanism underlying the changes in atrial SK2 and SK3 channels in diabetes.

PKC is extensively distributed in a variety of cardiac cells, including cardiomyocytes, endothelial cells, and smooth muscle cells. As a member of the serine threonine protein kinase family, it has several isomers and plays an important role in normal signal transduction, especially in the dynamic regulation of the plasma membrane. Once activated, PKC exerts a variety of effects and plays a role in myocardial differentiation, proliferation, growth, and apoptosis as well as in myocardial cell signaling [8, 33]. As an important member of the mitogen-activated protein kinase (MAPK) family, ERK can be activated by various stimuli and is widely involved in the regulation of cell functions, such as proliferation, differentiation, and apoptosis [34]. ERK is one of the downstream molecules of PKC, and the PKC/ERK pathway is closely related to the inflammatory response, collagen synthesis, and apoptosis [7]. Once activated, PKC phosphorylates and activates ERK, causing an inflammatory response, inhibiting apoptosis, promoting cell proliferation, and resulting in injury [35].

Several in vitro and in vivo non-ion channel studies have shown that diabetes-related hyperglycemia, insulin resistance, inflammatory cytokines, and oxidative stress can activate PKC and ERK [9, 10, 36–38]. Metformin reduces the oxidative stress, inflammatory response, and apoptosis by inhibiting the activation of PKC and ERK [11, 12, 39, 40]. Further research on ion channels showed that the phosphorylation of PKC plays an important role in the pathophysiology of ion channels. Cao et al. reported that PKC was involved in regulating the opening of myocardial large conductance Ca^2+^-activated K^+^ channels (BKCa) [41]. A study by Chahine showed that PMA (a PKC agonist) reduced the opening time and extended the shut-off time of alpha1D Ca*2+* channel current at the whole cell level and concluded that this may induce AF [42]. Puglisi et al. found that the activation of PKC reduced the outward ion currents and increased the inward ion currents in cardiomyocytes, thereby extending the APD [43]. In addition, SK channels have specific PKC phosphorylation sites [44]. We believe that the activation or inhibition of PKC will inevitably affect the expression of SK channels on the myocardial cells. However, we cannot rule out a more comprehensive effect of PKC. A report by Buchanan showed that the activation of PKC inhibited the SK ion current of Schaffer collateral synapses [45]. Sterling et al. demonstrated that PKC activation mediated the internalization of SK channels in renal cortical collecting duct cells [46]. Besides, several in vitro studies reported that intracellular ERK signaling pathway was involved in the regulation of ion current and expression of BKCa [47, 48].

In this study, we hypothesized and verified that metformin regulates SK2 and SK3 protein expression in the atrium of T2DM rats through the PKC/ERK signaling pathway. We infer that metformin regulates the expression and function of SK2 and SK3 channels and prevents or reduces diabetic arrhythmias by inhibiting the activation of the PKC/ERK signaling pathway, directly acting on the PKC phosphorylation sites of SK channels and working through indirect mechanisms, such as inhibiting oxidative stress, reducing the inflammatory response, and regulating transcription factors.

### Limitations

The current study also has some limitations. First, the overall activity of PKC in rat atrium but not the activity of each PKC isoform was measured in this study, while selective targeting of the specific isoform involved in SK regulation may be better to improve AF treatment in diabetic patients. Thus, further research is needed to investigate the effect of each PKC isoform on SK channels. Second, the respective roles and interactions of SK2 and SK3 in T2DM atrium need to be further clarified. Third, the PKC/ERK signaling pathway is an ubiquitous signaling pathway in cells, and metformin may mediate its effects through other mechanisms in addition to modulating the PKC/ERK signaling pathway. Thus, the deeper signaling mechanisms that metformin regulates SK2 and SK3 channels are still needed to be demonstrated by further studies.

## Conclusions

**Fig. 5:**
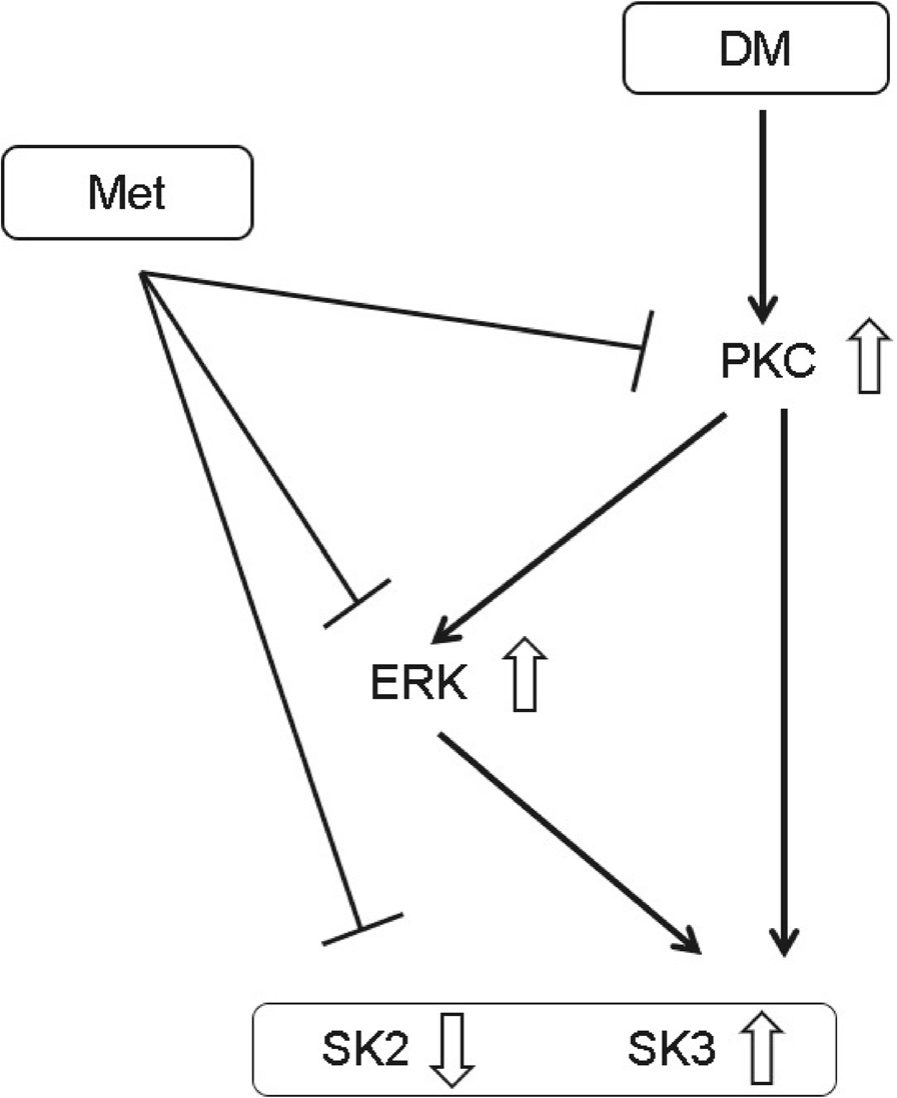
A proposed pathway for the protective effects of metformin against DM-induced disorders on atrial SK2 and SK3 channels. DM induces an excessive activitiy of PKC which is followed by a sequential activation of ERK and causes disorders of atrial SK2 and SK3 channels. Metformin treatment leads to a sequential inhibition on the activities of PKC and ERK and results in an improvement on the disorders

The results of this study indicate that the PKC/ERK signaling pathway is involved in the downregulation of SK2 and the upregulation of SK3 in the atrium of T2DM rats and long-term metformin treatment prevents the SK2 downregulation and the SK3 upregulation through inhibiting the PKC/ERK signaling pathway. Based on the findings, we proposed a hypothetical pathway for the metformin-mediated protection against the DM-induced disorders on atrial SK2 and SK3 channels (Fig. 5). Although there are other components needed to be validated in this signaling pathway, these results nonetheless provide a new insight into atrial electric remodeling in T2DM and metformin prophylaxis for diabetic AF as well as a theoretic basis for the selection of specific ion channel targets for antiarrhythmic drugs.

## Additional file

Additional file 1: **Table S1.** Raw data of relative PKC activity. **Table S2.** Raw data of Western blotting detection of SK2 expression. **Table S3.** Raw data of Western blotting detection of SK3 expression. **Table S4** Raw data of Western blotting detection of pERK expression. **Table S5.** Raw data of mean optical density values of SK2 expression. **Table S6.** Raw data of mean optical density values of SK3 expression. (ZIP 25 kb)

## Abbreviations

AF: Atrial fibrillation
ERK: Extracellular signal-regulated kinase
FBG: Fasting blood glucose
FINS: Fasting insulin
Met: Metformin
PKC: Protein kinase C
PMA: Phorbol 12-myristate 13-acetate
rh-EGF: Recombinant human epidermal growth factor
SK channels: Small conductance calcium-activated potassium channels
STZ: Streptozotocin
T2DM: Type 2 diabetes mellitus
